# Nutritional and herbal supplements for anxiety and anxiety-related disorders: systematic review

**DOI:** 10.1186/1475-2891-9-42

**Published:** 2010-10-07

**Authors:** Shaheen E Lakhan, Karen F Vieira

**Affiliations:** 1 Global Neuroscience Initiative Foundation, Los Angeles, CA, USA

## Abstract

**Background:**

Over the past several decades, complementary and alternative medications have increasingly become a part of everyday treatment. With the rising cost of prescription medications and their production of unwanted side effects, patients are exploring herbal and other natural remedies for the management and treatment of psychological conditions. Psychological disorders are one of the most frequent conditions seen by clinicians, and often require a long-term regimen of prescription medications. Approximately 6.8 million Americans suffer from generalized anxiety disorder. Many also suffer from the spectrum of behavioural and physical side effects that often accompany its treatment. It is not surprising that there is universal interest in finding effective natural anxiolytic (anti-anxiety) treatments with a lower risk of adverse effects or withdrawal.

**Methods:**

An electronic and manual search was performed through MEDLINE/PubMed and EBSCO. Articles were not discriminated by date of publication. Available clinical studies published in English that used human participants and examined the anxiolytic potential of dietary and herbal supplements were included. Data were extracted and compiled into tables that included the study design, sample population, intervention, control, length of treatment, outcomes, direction of evidence, and reported adverse events.

**Results:**

A total of 24 studies that investigated five different CAM monotherapies and eight different combination treatments and involved 2619 participants met the inclusion criteria and were analyzed. There were 21 randomized controlled trials and three open-label, uncontrolled observational studies. Most studies involved patients who had been diagnosed with either an anxiety disorder or depression (n = 1786). However, eight studies used healthy volunteers (n = 877) who had normal levels of anxiety, were undergoing surgery, tested at the upper limit of the normal range of a trait anxiety scale, had adverse premenstrual symptoms or were peri-menopausal, reported anxiety and insomnia, or had one month or more of elevated generalized anxiety. Heterogeneity and the small number of studies for each supplement or combination therapy prevented a formal meta-analysis. Of the randomized controlled trials reviewed, 71% (15 out of 21) showed a positive direction of evidence. Any reported side effects were mild to moderate.

**Conclusions:**

Based on the available evidence, it appears that nutritional and herbal supplementation is an effective method for treating anxiety and anxiety-related conditions without the risk of serious side effects. There is the possibility that any positive effects seen could be due to a placebo effect, which may have a significant psychological impact on participants with mental disorders. However, based on this systematic review, strong evidence exists for the use of herbal supplements containing extracts of passionflower or kava and combinations of L-lysine and L-arginine as treatments for anxiety symptoms and disorders. Magnesium-containing supplements and other herbal combinations may hold promise, but more research is needed before these products can be recommended to patients. St. John's wort monotherapy has insufficient evidence for use as an effective anxiolytic treatment.

## Background

Mental disorders plague millions of people around the world. Depression and anxiety are two of the most common mental disorders, affecting nearly 55 million people in the United States alone [1]. The complexities of the central nervous system make diagnoses, treatment, and amelioration of these debilitating illnesses exceptionally difficult. Advancement in these areas would be invaluable contributions in the effort to reduce the global impact of anxiety-based conditions. The universality of herbal remedies in many cultures makes them an appropriate treatment to explore.

According to the Diagnostic and Statistical Manual of Mental Disorders (DSM-IV-TR), anxiety is characterized by a feeling of persistent worry that hinders an individual's ability to relax [2]. This can range from the transient anxiety levels a person feels before surgery or a menstrual cycle to the pervasive feeling of nervousness that characterizes an anxiety disorder (e.g. generalized anxiety disorder, obsessive-compulsive disorder, panic disorder and social phobia). The impact of the anxiety is not limited to consistent stress, which is associated with higher risk of cardiovascular and cerebrovascular disease [3]. Anxiety also has debilitating physical manifestations as headaches, uncontrolled trembling and sweating, muscle tension and aches, among others.

To date, the biological explanations for many types of anxiety disorders remain inadequate. Postulations have implicated a dysregulation of specific neurotransmitters such as serotonin, dopamine and gamma-aminobutyric acid (GABA) as potential causes for both depression and anxiety disorders [4-6]. These hypotheses are based on the results of pharmacological treatments, but there are no definitive clinical trials that demonstrate the dysregulation of these neurotransmitters as causative factors of anxiety, potentially explaining why the treatment of anxiety with antidepressants is often ineffective. Thus far, cognitive behavioural therapy (CBT) has proven to be the most effective, long-term treatment for anxiety-related disorders [7].

With the lifetime prevalence of anxiety disorders reaching 16.6% worldwide [8], great strides have been made with ongoing research into its causes and treatments. In addition to antidepressants, serotonin-specific reuptake inhibitors (SSRIs) and benzodiazepines have also been prescribed to patients suffering from GAD [9,10]. However, while often effective, both of these classes of drugs come with many unwanted side effects such as suicidal ideation, decreased alertness, sexual dysfunction and dependency [11-16]. Additionally, the costs of these medications pose problems to patients who must take them on a daily, long-term basis

As a result, there has been increased interest in the use of complementary and alternative medicines (CAM) as a natural method for treating numerous types of anxiety. Herbs such as passionflower, kava, St. John's wort and valerian root, as well as the amino acid lysine and the cation magnesium, have been used for centuries in folk and traditional medicine to calm the mind and positively enhance mood. However, the efficacy and safety of utilizing CAMs to treat anxiety, both as a symptom and as a disorder, has only just begun to be rigorously tested in clinical trials within the last 10 to 15 years [17-19].

A number of reviews of the clinical effectiveness of herbal and nutrient treatments for depression, anxiety disorders, and sleep disturbance have been published over the past decade [19-25]. These have reviewed data associated with a number of treatments, including St. John's Wort, S-adenosyl-methionine (SAM-e), B vitamins, inositol, choline, kava, omega-3 fatty acids/fish extracts, valerian, lavender, melatonin, passionflower, skullcap, hops, lemon balm, black cohosh, ginkgo biloba, extracts of Magnolia and Phellondendron bark, gamma-aminobutyric acid (GABA), theanine, tryptophan and 5-hydroxytryptophan (5-HTP). However, none of these studies has been conducted in a systematic way.

The objective of this paper is to systematically review and summarize the available literature on herbal remedies and dietary supplements for treating anxiety and related symptoms in order to aid mental health practitioners in advising their patients and provide insight for future research in this field.

## Methods

### Search strategy

MEDLINE/PubMed and EBSCO databases were searched without regard for date of publication, using the search terms "alternative therapies," "herbal supplement" and individual herb and supplement names from popular sources, each crossed with the term "anxiety." In addition, key publications were hand-searched for references. [See Additional file 1 for a Quality of Reporting of Meta-analyses (QUOROM) statement checklist.]

### Selection criteria

The search was restricted to herbs and supplements that acted as anxiolytic agents and whose effects were measured either through quantitative rating scales or self-reports. Studies also had to be published in English, conducted with human subjects, have a sample size greater than 10, use a whole extract of the plant (if applicable) and detail data clearly. Case studies, review articles, meta-analyses, safety trials and studies that attempted to link vitamin and mineral deficiencies to the presence or absence of anxiety symptoms were excluded, as were trials in animals. Studies of anxiety parameters in healthy volunteers were also examined to provide supporting evidence.

### Data abstraction and synthesis of results

Study results were abstracted into data tables (Tables 1, 2, 3. 4, 5, 6). Because of the heterogeneous nature of the patients, preparations and outcome measures, data pooling was not possible. Therefore, the data was summarized qualitatively. The most common outcome measures encountered in these trials included: Hamilton Anxiety Scale (HAMA), State Trait Anxiety Inventory (STAI), Erlanger Anxiety, Tension and Aggression Scale (EAAS), Bf-S self-rating scale of well-being, Anxiety Sensitivity Index (ASI), and Clinical Global Impressions (CGI) scale. Some studies used measurements of anxiety biomarkers such as adrenocorticotropic hormone, cortisol, adrenaline, noradrenaline and chromogranin-A levels; skin conductance; heart rate; and blood pressure. A significant positive change in at least one of the primary outcome measures was required to categorize the trial as positive.

**Table 1:** Participant characteristics

	Passionflower	Kava	St. John's wort	Lysine	Magnesium	All studies
**Patients (n)**	278	1054	762	137	388	2619

**Gender**						

*Male*	46 (17%)	227 (22%)	246 (32%)	83 (61%)	130 (34%)	732 (28%)

*Female*	50 (18%)	759 (72%)	516 (68%)	54 (39%)	258 (66%)	1637 (63%)

*Not Reported*	182 (65%)	68 (6%)	-	-	-	250 (9%)

**Age range (years)**	19-47	18-75	18-65	20-59	18-82	18-82

**Race/Ethnicity**						

*Asian*	-	2 (< 1%)	-	108 (79%)	-	110 (4%)

*Caucasian*	-	401 (38%)	83 (11%)	29 (21%)	-	513 (20%)

*African American*	-	14 (1%)	-		-	14 (1%)

*Hispanic*	-	7 (< 1%)	-		-	7 (< 1%)

*Native American*	-	7 (< 1%)	-		-	7 (< 1%)

*Not Reported*	278 (100%)	623 (59%)	679 (89%)		316 (100%)	1896 (72%)

**Table 2:** Trials testing passionflower

Reference	Study Design	Sample Population	Intervention	Control	Length of Treatment	Outcomes	Direction of Evidence	Reported Adverse Events
Bourin (1997) [34]	Randomized; Double-blind; Parallel Group	182 outpatients with adjustment disorder with anxious mood	Euphytose^1^; 2 tablets, 3 times a day	Placebo tablets	28 days	Significant reduction in HAMA scores (from D7 to D28) in favour of Euphytose treatment	+	No serious AEs. Dry mouth Headache Constipation Drowsiness

Akhondzadeh (2001) [32]	Randomized; Double-blind; Parallel group	36 outpatients with DSM-IV for GAD for at least 6 months	45 drops/day of Passiflora extract plus placebo tablet	Oxazepam 30 mg/day plus placebo drops	4 weeks	Decrease in HAMA for both treatments^2^; overall no significant difference in efficacy between treatments	+	Higher impairment of job performance in oxazepam group; overall no significant difference in total side effects^3^

Movafegh (2008) [33]	Randomized; Double-blind; Parallel Group	60 patients undergoing inguinal herniorrhaphy	Oral Passiflora incarnata (500 mg, Passipy™ IranDarouk)	Placebo	Given as pre-medication 90 minutes before surgery	NRS anxiety scores were significantly lower in the passiflora group	+	Not reported

AEs: Adverse events; HAMA: Hamilton Anxiety Scale; DMS-IV: Diagnostic and Statistical Manual of Mental Disorders, fourth edition; GAD: generalized anxiety disorder; NRS: numerical rating scale.

1. Combination of *Crataegus oxyacantha *(10 mg), *Ballota foetida *(10 mg), *Passiflora incarnata *(40 mg), *Valeriana officinalis *(50 mg), *Cola nitida *(15 mg) and *Paullinia cupana *(15 mg).

2. D4 oxazepam; D7 passiflora.

3. Passiflora, mild/moderate: Dizziness, Drowsiness, Confusion, Ataxia, Allergic reaction, Impairment of job performance.

**Table 3:** Trials testing kava

Reference	Study Design	Sample Population	Intervention	Control	Length of Treatment	Outcomes	Direction of Evidence	Reported Adverse Events
Volz (1997) [42]	Randomized; Double-blind; Parallel Group	101 outpatients with anxiety of non-psychotic origin^1^	Kava-kava extract WS 1490 (90- 110 mg dry extract = 70 mg kl per capsule)	Placebo	24 weeks	Significant reduction in anxiety (HAMA, CGI, SCL-90-R, AMS) in favour of kava-kava treatment.	+	Excellent tolerability, similar to placebo; no clinically relevant changes in laboratory results. Stomach upset.

Scherer (1998)* [48]	Open-label; Uncontrolled Observational study	52 outpatients with nonpsychotic anxiety	Kava preparation (no dose reported in abstract)	N/A	Not reported in abstract	42 patients (80.8%) rated kava treatment as "very good" or "good".	+	Rare

Malsch (2001) [45]	Randomized; Double-blind; Parallel group	40 adult outpatients with non-psychotic nervous anxiety, tension and restlessness, impairing work performance, normal social activities and relationships^2^	Pre-treatment with benodiazepines (tapered off over two weeks) followed by capsules of 50 mg/day of dry extract standardized to 35 mg kava lactone for three weeks	Pre-treatment with benodiazepines (tapered off over two weeks) followed by placebo for three weeks	5 weeks	Significant reduction in anxiety (HAMA, Bf-S, EAAS, CGI) in kava-treated group.	+	No serious adverse events

Watkins (2001) [44]	Randomized; Double-blind; Parallel Group	13 patients with GAD	Kava 280 mg/day (standardized to 30% kavalactones)	Placebo	4 weeks	Significant improvement in baroreflex control of heart rate in kava-treated group; respiratory sinus arrhythmia did not respond to kava treatment.	+	Not reported

Connor (2002) [52]	Randomized; Double-blind; Parallel Group	38 adults with DSM-IV GAD^3^	Kava (standardized to 70 mg kavalactones [kl]). Treatment initiated at 149 mg kl/day and increased to 280 mg kl/day for the next 3 weeks.	Placebo	4 weeks	No significant difference to placebo^4^	-	Well tolerated. No evidence of withdrawal or sexual side effects.

Boerner (2003) [43]	Randomized; Double-blind; Parallel Group	129 outpatients diagnosed with GAD (GAD; ICD-10: F41.1)	400 mg/day Kava extract LI 150 (standardized to 30% kavapyrones, extraction solvent 96% ethanol in water, drug-extract ratio 13-20:1)	(1) 10 mg/day Buspirone or (2) 100 mg/day Opipramol	8 weeks	Kava was shown to be as effective as reference treatments; 75% of patients responded (50% reduction of HAMA score).	+	1 treatment-related adverse event. No systematic difference between treatments. No liver toxicity reported^5^.

Cagnacci (2003) [46]	Randomized; Open; Parallel Groups (3)	80 peri-menopausal women	Calcium (1 g/day) plus: (1) Kava-Kava,100 mg/day (55% of kavaina; Natural Bradel, Milano, Italy) (2) Kava-Kava, 200 mg/day	Calcium (1 g/day)	3 months	Significant reduction in STAI scores in favour of combination treatment.	+	Mild/moderate: Nausea Gastric pain. No liver toxicity.

Gastpar (2003) [50]	Randomized; Double-blind; Parallel Group	141 adult outpatients diagnosed with neurotic anxiety^6 ^	150 mg/day kava special extract WS 1490 (standardized to 35 mg kl)	Placebo	4 weeks	Pronounced decrease in ASI score for the kava group; however not statistically significant overall; however an exploratory analysis of variance across the differences between treatment end and baseline, with center as a second factor, showed superiority of kava over placebo.	-	Increased tiredness. No liver toxicity

Jacobs (2005) [53]	Randomized; Double-blind; Parallel Group (3)	391 healthy volunteers with anxiety^7 ^and insomnia	(1) 100 mg kl/day kava (30% total kavalactones in extract) with valerian placebo (2) 6.4 mg/day valerian (1% valerenic acid in extract) with kava placebo	Double placebo	28 days	Greater reductions in placebo group, but not statistically significant (STAI-State substest).	-	Similar frequency between treatments and placebo. No reports of liver toxicity

Sarris (2009) [47]	Randomized; Double-blind; Crossover	41 adult participants with 1 month or more of elevated generalized anxiety	Kava tablets (250 mg/day kavalactones)	Placebo	3 weeks	Highly significant reduction in anxiety (HAMA, BAI, MADRS) in kava-treated group.	+	No serious adverse events. Mild dizziness, nausea. No liver toxicity.

Sarris (2009) [51]	Randomized; Double-blind; Crossover	28 adults with MDD and co-occurring anxiety	Hypericum perforatum^8^ (1 × 1.8 g tablet, three times/day); Kava rhizome aqueous extract^9^ (1 × 2.66 g tablet, 3 times/day)	Placebo	4 weeks	Combination treatment had no significant effects on anxiety (BDI-II).	-	No serious adverse events. Mild gastrointestinal upset. No liver toxicity

HAMA: Hamilton Anxiety Scale; CGI: Clinical Global Impressions; SCL-90-R-ANX: Self-Report Symptom Inventory-90 Items revised, subscore somatic anxiety; AMS: Adjective Mood Scale; kl: kavalactones (kl); Bf-s: Befindlichkeitsskala [subjective well-being score]; EAAS: Erlanger Anxiety, Tension and Aggression Scale; DSM-IV: Diagnostic and Statistical Manual of Mental Disorders, fourth edition; GAD: generalized anxiety disorder; BAI: Beck Anxiety Inventory; BDI-II: Beck Depression Inventory-II; NADRS: Montgomery-Asberg Depression Rating Scale; MDD: major depressive disorder; SARA: Self-Assessment of Resilience and Anxiety; HADS: Hospital Anxiety and Depression Scale.

* No full text available.

1. DSM-III-R criteria: agoraphobia, specific phobia, generalized anxiety disorder and adjustment disorder with anxiety.

2. Diagnosis of agoraphobia (300.22), simple (300.29) or social phobia (300.23), generalized anxiety disorders (300.02) or adaptation disturbances (309.24) according to DSM-III-R.

3. According to the Results section, "Thirty-eight subjects were randomized, including 31 female (82%) and 32 Caucasian participants (97%)...Three subjects withdrew their consent following the baseline visit...and did not return for further assessment, leaving 35 subjects in the evaluable sample;" however, the Abstract states: "Thirty-seven adults with DSM-IV GAD were randomly assigned to...treatment."

4. Post-hoc analyses: kava was superior in low anxiety (SARA) and placebo was superior in high anxiety (HADS; SARA)

5. Slight increases in transaminase levels to above the upper limit of normal were reported in all three groups.

6. DSM-III-R diagnoses 300.02, 300.22, 300.23, 300.29, or 309.24.

7. Scores of at least 0.5 standard deviations above the mean on STAI-State.

8. Standardized to 990 μg of hypericin, and 1500 μg of flavone glycosides.

9. Standardized to 50 mg of kavalactones.

**Table 4:** Trials testing St. John's wort

Reference	Study Design	Sample Population	Intervention	Control	Length of Treatment	Outcomes	Direction of Evidence	Reported Adverse Events
Taylor (2000) [63]	Open-label; Uncontrolled; Observational	13 subjects with a primary DSM-IV diagnosis of OCD of at least 12 month duration	Fixed dose of 900 mg/day of 0.3% hypericin (a psychoactive compound in Hypericum)	N/A	12 weeks	Significant improvement in Y-BOCS scores in SJW group (comparable to those seen in clinical trials with SSRIs).	+	Diarrhea Restless sleep

Volz (2002) [61]	Randomized; Double-blind; Parallel Group	149 outpatients diagnosed with somatization Disorder^2^, undifferentiated somatoformDisorder^3^, or somatoform autonomic Dysfunctions^4 ^	Hypericum extract LI 160 (600 mg/day)	Placebo	6 weeks	Significant reduction in anxiety (HAMA-SOM, CGI, HAMA-T, HAMA-PSY, HDS, SCL-90-R, SCL-90-R-ANX) in favour of SJW treatment.	+	Verywell tolerated. Mild/moderate: Abdominal pain Arthritis Arrythmia Bronchitis Cystitis Headache Neuralgia

Muller (2003) [62]	Open-label; uncontrolled observational	500 patients diagnosed with depression comorbid with anxiety	(1) 500 mg valerian extract^5 ^and 600 mg/day St John's Wort^6^(2) 1,000 mg valerian extract^7 ^and 600 mg/day St John's wort^6^	N/A	6 weeks	Significant reduction in anxiety disorder symptoms (HAMA) in both treatment groups. Higher dosage results in greater improvements.	+	Allergy Bad dreams Sleep disorders Dysphoria

Kobak (2005) [60]	Randomized; Double-blind;Parallel Group	40 subjects with GAD	St John's wort^8^; flexible dose (600-1800 mg/day), mean dose at week 12 was 1676 mg/day	Placebo	12 weeks	No significant difference to placebo (LSAS)	-	Similar to placebo. Mild/moderate: Gastrointestinal upset Dizziness Insomnia Fatigue

Kobak (2005) [59]	Randomized; Double-blind; Parallel Group	60 outpatients with primary diagnosis of OCD	St John's wort LI 160^8^; flexible dose (600-1800 mg/day), mean dose at week 12 was 1663 mg/day	Placebo	12 weeks	No significant difference to placebo (Y-BOCS)	-	Similar to placebo^9^. Mild/moderate: Headache Gastrointestinal symptoms Fatigue Agitation Sleep disturbance

Sarris (2009) [51]	Randomized; Double-blind; Crossover	28 adults with MDD and co-occurring anxiety	Hypericum perforatum^10^ (1 × 1.8 g tablet, three times/day); Kava rhizome aqueous extract^11^(1 × 2.66 g tablet, 3 times/day)	Placebo	4 weeks	Combination treatment had no significant effects on anxiety (BDI-II).	-	No serious adverse events. Mild gastrointestinal upset. No liver toxicity

DSM-IV: Diagnostic and Statistical Manual of Mental Disorders, fourth edition; Y-BOCS: Yale-Brown Obsessive-Compulsive Scale; OCD: obsessive-compulsive disorder; GAD: generalized anxiety disorder; SJW: St John's wort; SCL-90-R-ANX: Self-Report Symptom Inventory-90 Items revised, subscore somatic anxiety; HAMA: Hamilton Anxiety Scale; ICD-10; International Classification of Diseases; LSAS: Liebowitz Social Anxiety Scale; HAMA-SOM: Hamilton Anxiety Scale, subscore somatic anxiety; CGI: Clinical Global Impressions; HAMA-PSY: Hamilton Anxiety Scale, subscore psychic anxiety; HAMA-T: Hamilton Anxiety Scale, total score; HDS: Hamilton Depression Scale; SCL-90-R: Self-Report Symptom Inventory-90 Items revised; PGI-I: Patient Global Impressions of Improvement; CGI-I: Clinical Global Impressions of Improvement; CGI-S: Clinical Global Impressions of Severity.

1. Clinician observation [case 1], SCL-90-R [case 2]; self-assessment, HAMA [case 3].

2. ICD-10: F45.0.

3. F45.1.

4. F45.3.

5. Euvegal Balance tablet; drug-extract-ratio 3-6:1.

6. Neuroplant tablet; drug-extract-ratio 2.5-5:1.

7. Euvegal Balance tablet.

8. Drug/extract ratio of 3-6:1.

9. Except agitation which was higher with SJW.

10. Standardized to 990 μg of hypericin, and 1500 μg of flavone glycosides.

11. Standardized to 50 mg of kavalactones.

**Table 5:** Trials testing lysine

Reference	Study Design	Sample Population	Intervention	Control	Length of Treatment	Outcomes	Direction of Evidence	Reported Adverse Events
Jezova (2005) [67]	Randomized; Double-blind; Parallel Group	29 healthy male subjects at the upper limit of the normal range of a trait anxiety scale^1^	Mixture of L-lysine and L-arginine (3 g each/day)	Placebo	10 days	AMino acid treatment enhanced adrenocorticotropic hormone, cortisol, adrenaline and noradrenaline levels and galvanic skin responses during stress; no effect on heart rate and blood pressure.	**+**	None

Smriga (2007) [68]	Randomized; Double-blind; Parallel Group	108 healthy Japanese adults	Oral L-lysine (2.64 g/day) and L-arginine (2.64 g/day)	Placebo	1 week	L-lysine/L-arginine treatment significantly reduced trait and state anxiety; also decreased basal levels of salivary cortisol and chromogranin-A in male subjects	**+**	None

STAI: State Trait Anxiety Inventory.

1. Scored above 45 on STAI questionnaire.

**Table 6:** Trials testing magnesium

Reference	Study Design	Sample Population	Intervention	Control	Length of Treatment	Outcomes	Direction of Evidence	Reported Adverse Events
Carroll (2000) [75]	Randomized; Double-blind; Parallel Group	80 healthy males	Berocca: oral multivitamin^1^	Placebo	28 days	Multivitamin treatment significantly reduced anxiety as measured by GHQ-28, HADS and PSS.	+	Not reported

De Souza (2000) [76]	Randomized; Double-blind; Crossover (4)	44 women with adverse premenstrual symptoms but otherwise in good health	(1) 200 mg Mg, (2) 50 mg vitamin B_6_, (3) 200 mg Mg + 50 mg vitamin B_6 _per day	Placebo	One menstrual cycle	200 mg/day Mg + 50 mg/day vitamin B_6 _significantly reduced anxiety-related premenstrual symptoms	+	Participants were not specifically asked, but none were reported spontaneously

Hanus (2004) [77]	Randomized; Double-blind; Parallel Group	264 patients with generalized anxiety (DSM-III-R) of mild-to-moderate intensity^2^	Sympathyl: extracts of *crataegus oxyacantha *and *eschscholtzia californica *plus magnesium	Placebo	3 months	Significant clinical improvement in anxiety^3 ^in favour of the combination treatment	+	No serious AEs related to treatment^4^

GHQ-28: General Health Questionnaire; HADS: Hospital Anxiety and Depression Scale; PSS: Perceived Stress Scale; DMS-II-R: Diagnostic and Statistical Manual of Mental Disorders, third edition revised; HAMA: Hamilton Anxiety Scale; HAMA-SOM: Hamilton Anxiety Scale, subscore somatic anxiety; HAMA-T: Hamilton Anxiety Scale, total score (HAMA-T

1. Multivitamin containing vitamin B1 (15 mg), B2 (15 mg), niacin (50 mg), pantothenic acid (23 mg), B6 (10 mg), biotin (150 mcg), folic acid (400 mcg), B12 (10 mcg), C (500 mg), calcium (100 mg), magnesium (100 mg), zinc (10 mg).

2. Total HAMA score between 16 and 28.

3. Measured by HAMA-T and HAMA-SOM and subjective patient-rated anxiety.

Headache, muscular stiffness, insomnia, drowsiness, indifference, anxiety, palpitations, nausea (4), gastralgia, diarrhea, gastric heaviness, dysuria, colic renal pain, morning sluggishness (3), asthenia.

## Results and discussion

### Flow of included studies

**Figure 1:**
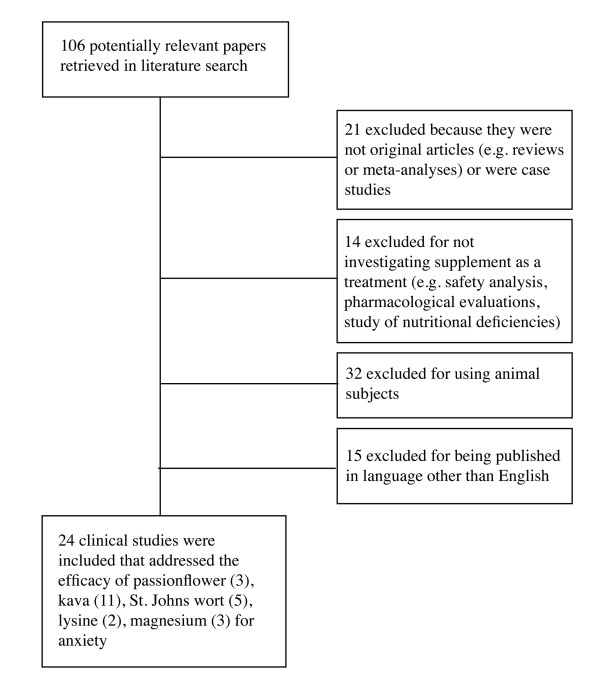
**Flow diagram of included studies**.

Electronic searches found 106 papers that were potentially relevant to the present systematic review. Of these, 24 met the inclusion/exclusion criteria (see Figure 1 for a flow diagram). Of the 82 that did not meet the criteria, 21 were excluded from the main review because they were not original research (e.g. reviews or meta-analyses) or were case studies, 14 did not investigate the supplement as a treatment (e.g. safety analysis, pharmacological evaluations, study of nutritional deficiencies), 32 did not use human subjects, and 15 were published in a language other than English. Some of the excluded papers listed as reviews are cited in the background and discussion sections of this manuscript. Papers that were mainly discussions of philosophical and ethical issues were not reviewed at all.

### Study characteristics

A total of 24 studies were found that met the aforementioned requirements. These studies examined the effectiveness of five monotherapies (passionflower, lysine, magnesium, kava and St John's wort) and eight combination treatments (a herbal combination, multivitamin, L-lysine + L-arginine, magnesium + vitamin B_6_, herbal combination + magnesium, calcium + kava, St John's wort + kava, St John's wort + valerian). Of these studies, 13 were randomized controlled trials in outpatients with a DSM-IV-diagnosed disorder, and three were randomized controlled trials in patients with other types of anxiety (perimenopausal, menstrual, and pre-surgery). Five trials were done in healthy volunteers, three of which recruited healthy volunteers with high-normal anxiety levels. In addition, there were three uncontrolled observational studies.

Overall, 2619 participants between the ages of 18 and 82 took part in these studies. Twenty-eight percent were male, 63% were female and 9% did not have their gender reported (Table 1). Ethnicity/race, although an important demographic factor, was not reported in 72% of patients. As a result, it is difficult to draw any overarching conclusions from the results because these factors can significantly affect the potential for herbs to treat anxiety illnesses.

Some cultures have a greater preference for natural medicine than modern medicine, and therefore will likely exhibit positive results towards it. Because culture, gender, and age are potential confounding variables, efforts should be made to control for them in future studies.

This review presents the available evidence for passionflower, lysine, magnesium, kava and St John's wort, either alone or in combination. Methodological details and results of these trials are summarized in Tables 2, 3, 4, 5, 6. These tables are divided according to the treatment studied and include the reference, study design, sample population, intervention, control, outcomes, direction of evidence, and reported adverse events.

### Herbal Medicines

#### Passionflower

Passionflower or *Passiflora incarnata Linn*. has a long history of use as an anxiolytic agent in folklore and has been used by people all over the world to treat anxiety [26]. More importantly, several studies involving the biochemical makeup of passionflower have been conducted [27-29]. Between the 1970 s and 1990 s, passionflower was listed as an official plant drug by the pharmacopoeias of America, Britain, Germany, France, Switzerland, Egypt and India; its wide use has made it an acceptable treatment for restlessness and nervousness [30].

The anxiolytic effects of passionflower are well documented in mice [30,31]. However, one of the problems with herbal supplements is that plant material contains thousands of phytochemicals, making it challenging to pinpoint the specific biochemicals responsible for the anxiolytic properties. In other words, although herbal remedies often produce positive results, identifying the active ingredients can be difficult. Therefore, users of herbal remedies may be consuming ineffective or possibly toxic substances in addition to the active, anxiolytic ingredients. To date, three human trials have documented the efficacy of passionflower as a treatment for anxiety-related disorders [32-34].

One double-blind, placebo-controlled study analyzed the difference in efficacy between oxazepam, a prescription benzodiazepine used to treat chronic anxiety symptoms, and passionflower in patients (n = 36) who met the criteria for GAD [32]. The results showed no difference between the two anxiolytics with regard to the treatment of GAD, suggesting that passionflower is as effective as benzodiazepines in eliminating anxiety symptoms. Subjects from the passionflower group also reported lower job impairment performance than those in the benzodiazepine group; however, subjects in the benzodiazepine group reported a faster onset of symptom relief.

This anxiolytic effect was also seen in two other subsets of patients: those undergoing surgery (n = 60) who were treated with passionflower monotherapy [33], and those diagnosed with adjustment disorder with anxious mood (n = 182) who were treated with passionflower in combination with *crataegus oxyacantha*, *ballota foetida*, *valeriana officinalis*, *cola nitida *and *paullinia cupana *[34].

Mild adverse events were reported in only one study, including dizziness, drowsiness and confusion [32]. This preliminary evidence suggests that passionflower may have a role in the treatment of anxiety and warrants future research.

#### Kava

Kava is a drink that is prepared from the plant *Piper methysticum*. It has been consumed in many cultures because it is known to relieve anxiety, restlessness and insomnia for centuries [35,36]. Several studies in animals have also demonstrated the kava plant's abilities as an anxiolytic agent [37,38]. The attractiveness of kava is that it is anxiolytic but not sedative or mentally impairing, which are typical side effects caused by benzodiazepines [32]. The biochemical mechanism of kava's anxiolytic activity has been postulated to occur through enhanced ligand binding to GABA type A receptors, blockage of violated-gated sodium channels and calcium ion channels, norepinephrine and dopamine reuptake inhibition, and reversible inhibition of monoamine oxidase (MAO) B [see [39] for a review]. To note, the binding of kava extracts to several neurotransmitters such as GABA_A1_, dopamine D2 and the opiates (μ and δ), were demonstrated *in vitro *and in the rat brain [40,41].

The first randomized, placebo-controlled, double-blind study of kava for the treatment of patients who were diagnosed with anxiety disorder was conducted in 1997 [42]. The subjects (n = 101) were given either an extract of kava or a placebo for 25 weeks. The participants who were given the kava extract showed improvement in their primary and secondary anxiety symptoms based on the HAMA -- a method of patient self-reporting -- and a CGI, which was determined by physicians. Primary anxiety is described as the inability to regulate stress and anxiety since early childhood. Secondary anxiety, which develops later in life, can be caused by clinical disorders. The researchers concluded that when kava is used an anxiolytic alternative to benzodiazepines or tricyclic antidepressants, individuals typically suffer from less side effects.

These results were later supported by five other RCTs [43-47] and one uncontrolled, observational study [48]. These studies showed kava's therapeutic potential both as a monotherapy for patients with anxiety disorder [48], GAD [43,44,49], elevated generalized anxiety [47] and those being tapered off of benzodiazepines [45], as well as in combination with calcium for perimenopausal women [46].

However, four RCTs showed that kava alone or in combination with St John's wort is no more effective than placebo in reducing symptoms of anxiety [50-53]. Two of these studies showed no significant difference between kava treatment and placebo [51,53], while one found that placebo was actually better at reducing anxiety symptoms in patients with higher baseline anxiety scores [52]. According to the researchers, this could have been partly due to the study population. In this trial, patients were actively looking for alternative treatments for their GAD and were, therefore, highly motivated for kava treatment to produce an effect. This in turn could have influenced their response to treatment and led to an increased placebo effect. It is important to note that the sample size of this study was very small.

The last negative trial [50] was classified on the basis that it failed to meet its primary endpoint -- a significant reduction in anxiety based on the Zung Anxiety Scale from memory. However, an exploratory analysis of variance across the differences between treatment end and baseline, with the treatment center as a second factor, showed superiority of kava over placebo. According to the researchers, this variance between centers did not endanger the validity of their findings; however, it did reinforce the importance of standardizing ratings across participating centers in multi-center studies.

All of these trials also revealed that taking doses less than 400 mg/day does not cause serious side effects. This is important to note, especially since the U.S. Food and Drug Administration (FDA) published a consumer advisory warning in 2002 about the potential for severe liver damage from kava-containing supplements [54]. This potential, yet rare, risk of hepatitis, cirrhosis and liver failure led nations such as Canada and the United Kingdom to ban kava supplements. However, Teschke *et al. *reported in 2008, that owing to the fact that kava was considered to be well tolerated until 1998 when the first reported cause of hepatotoxicity occurred, these rare, but serious side effects may have occurred due to poor quality kava, as well as other risk factors such as overdose, prolonged therapy and co-medication [55].

Of the 435 clinical trial participants taking kava supplements in our review, some at high doses, no liver issues were reported. Therefore, the current review supports the conclusion that liver toxicity is indeed a rare side effect.

#### St John's wort

*Hypericum perforatum*, or St John's wort (SJW), is derived from the flowering tops of a perennial shrub. It has been used in traditional medicine for centuries to treat a wide range of disorders and is licensed in Germany to treat anxiety, depression and sleep disorders [56]. There are numerous hypotheses for its anxiolytic effects based on the binding affinity of at least 10 different extracts, including naphthodianthrones like hypericins, flavonoids, xanthones, and bioflavonoids, for adenosine, GABA_A_, GABA_B _and glutamine receptors, as well as the inhibition of monoamine oxidase-A and -B activity and synaptosomal uptake of serotonin, dopamine and noradrenaline (norepinephrine) [57]. Of these active ingredients, hypericin has been studied the most, and the amount present is generally used to standardize extracts.

SJW is probably most recognized for its use in depression. A meta-analysis published in 1996, showed that SJW was more effective than placebo in treating mild to moderate clinical depression [56]. Based on the author's recommendations, researchers began comparing the efficacy and safety profile of SJW against other routinely prescribed antidepressants. One trial conducted in Germany concluded that SJW was as effective as imipramine in treating mild to moderate depression (n = 324) [58].

Depression has been linked to anxiety, with many symptoms, panic attacks for example, overlapping between the two disorders. Little is known about the specific reasons for the link in the conditions; however, there may be as high as an 85% overlap with the diagnoses and many conventional treatment options are prescribed for both disorders. There has been little study of the effectiveness of SJW in treating anxiety disorders specifically, with only four RCTs [51,59-61] and two uncontrolled observational studies [62,63].

These published studies presented contradictory results. A small 12-week observational study (n = 13) of patients with OCD showed that SJW caused significant improvements, with results comparable to those seen in clinical trials with SSRIs [63]. However, a larger 12-week RCT (n = 60) showed no significant difference between patients treated with SJW (at doses higher than the observational study) or those treated with placebo [59]. Based on previous studies, OCD has one of the lowest placebo response rates of all of the anxiety disorders [64]. For this reason, these negative results were probably due to lack of response to SJW treatment rather than the high placebo response rates noted in the negative kava trials [59].

A second set of RCTs investigated the use of SJW combination treatments for depression with co-morbid anxiety. A combination of SJW and valerian was found to significantly reduce anxiety disorder symptoms; however, greater reductions were seen with higher doses of valerian (SJW doses remained constant between treatment groups), suggesting that valerian has more of an effect on symptoms [62]. A combination of SJW and kava was shown to have no significant effects on anxiety [51].

Finally, a RCT of 149 patients with depression with co-morbid anxiety, OCD and somatization disorder demonstrated that six weeks of treatment with SJW significantly reduced anxiety [61]. However, a RCT of 40 patients diagnosed with generalized social anxiety disorder found that SJW was no more effective than placebo in reducing anxiety symptoms [60]. In the discussion of this study, the researchers stated that a negative trial was conducted but speculated that minimum severity levels may be necessary for SJW to be effective in this patient population [60].

More research needs to be done using SJW in all the indications presented in this review in order to determine its effectiveness. However, the results point to a potential anxiolytic agent with a side effect profile similar to placebo. All of the side effects reported in the reviewed trials were mild to moderate and were most often cases of gastrointestinal upset, dizziness, sleep disturbances, and headaches.

### Nutritional Supplements

#### Lysine

It has long been postulated that the dysregulation of neurotransmitters may be a cause for anxiety. These neurotransmitters include GABA, serotonin, dopamine and norepinephrine [4-6]. Amino acids such as L-tyrosine and L-tryptophan are known precursors for specific neurotransmitters. Recent studies in animals have identified two other amino acids, L-lysine and L-arginine [65,66], which may influence neurotransmitters involved in stress and anxiety. L-lysine has been shown to act as a partial serotonin receptor 4 (5-HT_4_) antagonist, decreasing the brain-gut response to stress as well as decreasing blood cortisol levels [65]. Based on the results from animal studies, two placebo-controlled studies were conducted to analyze the effects of L-lysine-containing supplements in humans [67,68].

The first of these clinical trials was conducted in healthy male volunteers who were suffering from high-trait anxiety based on a STAI questionnaire [67]. Results from this study showed that L-lysine and L-arginine combination supplements improved participants' ability to handle induced stress through an increase in cortisol, while placebo had no reported improvement of anxiety symptoms. In the discussion, the researchers attributed the increase in cortisol to a previous stress hormone regulation deficiency. A previous report indicated that during moments of induced stress, an increase in cortisol levels, which is the typical reaction in healthy persons, does not increase in people with high-trait anxiety [69]. This dysregulation of cortisol may lead to augmented feelings of anxiousness when stress is induced.

The second RCT recruited 108 healthy Japanese individuals [68]. After one week of treatment with an oral L-lysine and L-arginine supplement, basal levels of salivary cortisol decreased in male subjects (n = 54) but not in females, presumably because these participants were not selected based on high-trait anxiety. Supplementation also resulted in significant reductions in state anxiety (a temporary condition characterized by apprehension, tension and fear about a specific situation or activity) and trait anxiety (a pre-set level of anxiety or a tendency to be anxious) in both males and females.

For the two available RCTs, it seems that the L-lysine + L-arginine combination effectively reduces anxiety scores with no reported side effects. Amino acid supplements may also help in balancing cortisol levels triggered by stress in both healthy individuals and those with high trait anxiety. However, more research needs to be conducted on both lysine combinations and monotherapy to confirm these results.

#### Magnesium

Magnesium is a positively charged ion, a cation, that is involved in many important molecular functions in the body and has been linked to anxiety-related disorders [70-74]. To date, three human trials have been conducted that test the anti-anxiety effects of increased magnesium intake in combination therapies [75-77], and all showed a positive direction of evidence.

In the first study, 28-day treatments with a multivitamin that contained large amounts of magnesium, zinc and calcium dramatically decreased psychological distress (according to the GHQ-28) compared to placebo, which worsened symptoms [75]. Results from the HADS also showed a decrease in anxiety for the treatment group. The effects became more pronounced as the multivitamin treatment progressed but could not be linked solely to magnesium supplementation.

A second study published in 2000 looked at the effects of magnesium and vitamin B_6 _supplementation on premenstruation-related anxiety [76]. The women were given 1) magnesium, 2) B_6_, 3) magnesium + B_6_, and 4) a placebo over four menstrual cycles, respectively. The average magnesium intake for this study was approximately 300 mg daily. The women were asked to keep a log of their symptoms and categorize them into six groups: anxiety, craving, depression, hydration, other, and total. The results showed that the combination of magnesium and B_6 _created a synergistic affect that provided women with the greatest relief from premenstrual anxiety. However, magnesium monotherapy was shown to provide results similar to placebo.

The third clinical study was conducted in 2004 and investigated the effects of three compounds in combination, including magnesium, versus placebo in patients diagnosed with GAD (n = 264) [77]. The researchers found that both the magnesium-containing supplement and the placebo drastically decreased anxiety systems based on HAMA, a personal assessment, and a physician's evaluation, hinting at a potential placebo effect for this treatment. Also, due to the fact that one of the herbal extracts contained in the preparation is closely related to the opium poppy, these effects may not have been due to the action of the magnesium.

Although the exact mechanism has yet to be determined, it appears magnesium supplementation is effective at treating anxiety and anxiety-related disorders when used in combination with other vitamins, minerals and herbal extracts. However, more research of magnesium monotherapy and its pharmacology is needed to determine whether magnesium itself possesses anxiolytic characteristics. Overall, available literature shows that magnesium-containing supplements are generally well-tolerated with very few reported side effects.

## Conclusions

Anxiety disorders are one of many common psychological ailments. Natural remedies have been used for centuries in many cultures to alleviate anxiety and its symptoms with surprising effectiveness. In Western cultures, however, research that proves the usefulness of medicinal herbs and natural substances has only begun to gain momentum over the past few decades. In addition, the absence of proper guidelines governing the production and use of vitamins, minerals, amino acids and herbs for medicinal purposes is also causing the clinical prescription of these natural treatments to lag behind in the United States.

Of the RCTs reviewed in this report, 71% (15 out of 21) showed a positive direction of evidence, and any reported side effects were mild to moderate. Based on this data, it appears that nutritional and herbal supplements are effective methods for treating anxiety and anxiety-related conditions without the risk of serious side effects. However, the effectiveness of each of the reviewed combinations and monotherapies has not been substantiated to the same degree.

Passionflower has been studied in three different RCTs, twice as a monotherapy and once as part of an herbal combination. All three of these studies showed a positive benefit for treatment with passionflower, providing good evidence of its effectiveness as an anxiolytic agent. However, since each of these studies was conducted in a different patient type, more research is needed to prove its efficacy in each indication.

Kava is the most researched supplement in this review with 11 different studies (10 RCTs and one observational). Of the RCTs of kava monotherapy, 63% (5/8) showed treatment significantly reduced anxiety symptoms in a variety of patient types. This provides good evidence for the use of kava in patients with GAD, non-psychotic anxiety and other anxiety-related disorders.

The evidence for St John's wort was mixed, with 50% (3/6) of the studies having positive results. However, the fact that only 1 out of the 4 RCTs had a positive direction of evidence and that the active treatment in this trial was a combination of SJW and valerian suggests that SJW monotherapy should not be recommended to patients suffering from anxiety disorders or other anxiety-related conditions.

For all three of the reviewed herbal supplements, more research needs to be done to establish the most effective dosage and to determine whether this varies between different types of anxiety or anxiety-related disorders. Furthermore, as 3 of the 4 herbal combinations showed positive results, future research should focus on determining whether herbal combinations are similarly or more effective than monotherapy as well as refining the type of herbs and dosages contained in combination supplements.

Combination nutritional supplements containing lysine or magnesium also appear to hold promise as treatments for anxiety symptoms and disorders. Both RCTs of L-lysine and L-arginine combinations demonstrated positive results, providing good but limited evidence of its usefulness as a treatment for anxiety.

The evidence for magnesium is mixed. Even though all three RCTs of magnesium-containing supplements had positive results, magnesium monotherapy was shown to be no different than placebo [76], raising the question of whether magnesium provides any anxiolytic benefits in combination or whether the results were based on the actions of the other nutrients/herbal extracts. However, this study was conducted in women with premenstrual anxiety rather than an anxiety disorder. Future research should focus on elucidating magnesium's mode of action in order to determine if it has anxiolytic properties and provides any synergistic effects when combined with other natural anxiolytic agents.

Herbal medicines hold an important place in the history of medicine, as most of our current remedies, and the majority of those to be discovered in the future, will contain phytochemicals derived from plants. While locating the active ingredients in herbal substances is pivotal to being able to produce effective supplements, understanding the quantity needed and potency of different ways of extracting and preparing the phytochemicals is vital to creating a standard measure of their effectiveness. In addition, the dangers of overconsumption and interactions with prescription medications and over-the-counter medications need to be further analyzed. This understanding of the standards for effective preparation further minimizes the chance of side effects from herbal medicines and helps to create an undisputable body of evidence for their effectiveness.

## List of abbreviations

AMS: Adjective Mood Scale; ASI: Anxiety Sensitivity Index; BAI: Beck Anxiety Inventory; BDI-II: Beck Depression Inventory-II; Bf-S: Befindlichkeitsskala [subjective well-being score]; CAM: complementary and alternative medicine (CAM); CGI: Clinical Global Impressions; CBT: cognitive behavioural therapy; CGI-I: Clinical Global Impressions of Improvement; CGI-S: Clinical Global Impressions of Severity; DSM-III-R: Diagnostic and Statistical Manual of Mental Disorders, third edition revised; DSM-IV: Diagnostic and Statistical Manual of Mental Disorders, fourth edition; EAAS: Erlanger Anxiety, Tension and Aggression Scale; FDA: U.S. Food and Drug Administration, GABA: gamma-aminobutyric acid; GAD: generalized anxiety disorder; GHQ-28: General Health Questionnaire; HADS: Hospital Anxiety and Depression Scale; HAMA: Hamilton Anxiety Scale; HAMA-PSY: Hamilton Anxiety Scale, subscore psychic anxiety; HAMA-SOM: Hamilton Anxiety Scale, subscore somatic anxiety; HAMA-T: Hamilton Anxiety Scale, total score; HCl: hydrochloric acid; HDS: Hamilton Depression Scale; ICD-10: International Classification of Diseases; kl: kavalactones (kl); LSAS: Liebowitz Social Anxiety Scale; MAO: monoamine oxidase; MADRS: Montgomery-Asberg Depression Rating Scale; MDD: major depressive disorder; NRS: numerical rating scale; OCD: obsessive-compulsive disorder; PGI-I: Patient Global Impressions of Improvement; PSS: Perceived Stress Scale; QUOROM: Quality of Reporting of Meta-analyses; RCT: randomized controlled trial; SARA: Self-Assessment of Resilience and Anxiety; SCL-90-R: Self-Report Symptom Inventory-90 Items revised; SCL-90-R-ANX: Self-Report Symptom Inventory-90 Items revised, subscore somatic anxiety; SJW: St John's wort; SSRI: Serotonin selective reuptake inhibitor; STAI: State Trait Anxiety Inventory; Y-BOCS: Yale-Brown Obsessive-Compulsive Scale.

## Competing interests

The authors declare that they have no competing interests.

## Authors' contributions

SEL and KFV participated in the preparation of the manuscript. All authors read and approved the final manuscript.

## Supplementary Material

Additional file 1 **QUOROM Statement checklist**. Click here for file

## References

[B1] Kessler RC Chiu WT Demler O Merikangas KR Walters EE Prevalence, severity, and comorbidity of 12-month DSM-IV disorders in the National Comorbidity Survey Replication Arch Gen Psychiatry 2005 62 617 627 10.1001/archpsyc.62.6.617 15939839 PMC2847357

[B2] Diagnostic and Statistical Manual of Mental Disorders 2000 4 Washington D.C.: American Psychiatric Association

[B3] Vogelzangs N Seldenrijk A Beekman AT van Hout HP de Jonge P Penninx BW Cardiovascular disease in persons with depressive and anxiety disorders J Affect Disord10.1016/j.jad.2010.02.112 PMC2964458 20223521

[B4] Christmas D Hood S Nutt D Potential novel anxiolytic drugs Curr Pharm Des 2008 14 3534 3546 10.2174/138161208786848775 19075730

[B5] D'Hulst C Atack JR Kooy RF The complexity of the GABAA receptor shapes unique pharmacological profiles Drug Discov Today 2009 14 866 875 10.1016/j.drudis.2009.06.009 19576998

[B6] Furmark T Neurobiological aspects of social anxiety disorder Isr J Psychiatry Relat Sci 2009 46 5 12 19728568

[B7] Ballon D Anxiety Disorders: An Information Guide 2008 Toronto, Canada: Centre for Addiction and Mental Health

[B8] Somers JM Goldner EM Waraich P Hsu L Prevalence and incidence studies of anxiety disorders: A systematic review of the literature Can J Psychiatry 2006 51 100 103 16989109 10.1177/070674370605100206

[B9] Davidson JR Pharmacotherapy of generalized anxiety disorder J Clin Psychiatry 2001 62 46 50 10.4088/JCP.v62n0110 11414551

[B10] Davidson JR First-line pharmacotherapy approaches for generalized anxiety disorder J Clin Psychiatry 2009 70 25 31 10.4088/JCP.s.7002.05 19371504

[B11] Cascade E Kalali AH Kennedy SH Real-World Data on SSRI Antidepressant Side Effects Psychiatry (Edgmont) 2009 6 16 18 19724743 PMC2719451

[B12] Gunnell D Saperia J Ashby D Selective serotonin reuptake inhibitors (SSRIs) and suicide in adults: meta-analysis of drug company data from placebo controlled, randomised controlled trials submitted to the MHRA's safety review BMJ 2005 330 385 10.1136/bmj.330.7488.385 15718537 PMC549105

[B13] Hall WD Lucke J How have the selective serotonin reuptake inhibitor antidepressants affected suicide mortality? Aust N Z J Psychiatry 2006 40 941 950 17054562 10.1080/j.1440-1614.2006.01917.x

[B14] Hu XH Bull SA Hunkeler EM Ming E Lee JY Fireman B Markson LE Incidence and duration of side effects and those rated as bothersome with selective serotonin reuptake inhibitor treatment for depression: patient report versus physician estimate J Clin Psychiatry 2004 65 959 965 10.4088/JCP.v65n0712 15291685

[B15] Lader M Tylee A Donoghue J Withdrawing benzodiazepines in primary care CNS Drugs 2009 23 19 34 10.2165/0023210-200923010-00002 19062773

[B16] O'Brien CP Benzodiazepine use, abuse, and dependence J Clin Psychiatry 2005 66 28 33 10.4088/JCP.v66n0104 15762817

[B17] Kinrys G Coleman E Rothstein E Natural remedies for anxiety disorders: potential use and clinical applications Depress Anxiety 2009 26 259 265 10.1002/da.20460 19123457

[B18] Garcia-Garcia P Lopez-Munoz F Rubio G Martin-Agueda B Alamo C Phytotherapy and psychiatry: bibliometric study of the scientific literature from the last 20 years Phytomedicine 2008 15 566 576 10.1016/j.phymed.2008.04.014 18583120

[B19] Saeed SA Bloch RM Antonacci DJ Herbal and dietary supplements for treatment of anxiety disorders Am Fam Physician 2007 76 549 556 17853630

[B20] Meeks TW Wetherell JL Irwin MR Redwine LS Jeste DV Complementary and alternative treatments for late-life depression, anxiety, and sleep disturbance: a review of randomized controlled trials J Clin Psych 2007 68 1461 1471 10.4088/JCP.v68n1001 17960959

[B21] Brown RP Gerbarg PL Herbs and nutrients in the treatment of depression, anxiety, insomnia, migraine, and obesity J Psychiatr Pract 2001 7 75 91 10.1097/00131746-200103000-00002 15990509

[B22] Weeks BS Formulations of dietary supplements and herbal extracts for relaxation and anxiolytic action: Relarian Med Sci Monit 2009 15 RA256 262 19865069

[B23] Ross BM Omega-3 polyunsaturated fatty acids and anxiety disorders Prostaglandins Leukot Essent Fatty Acids 2009 81 309 312 10.1016/j.plefa.2009.10.004 19906519

[B24] Geller SE Studee L Botanical and dietary supplements for mood and anxiety in menopausal women Menopause 2007 14 541 549 10.1097/01.gme.0000236934.43701.c5 17194961

[B25] Cauffield JS Forbes HJ Dietary supplements used in the treatment of depression, anxiety, and sleep disorders Lippincotts Prim Care Pract 1999 3 290 304 10711131

[B26] Dhawan K Kumar R Kumar S Sharma A Correct Identification of Passiflora incarnata Linn., a Promising Herbal Anxiolytic and Sedative J Med Food 2001 4 137 144 10.1089/109662001753165710 12639407

[B27] Boeira JM Fenner R Betti AH Provensi G Lacerda LD Barbosa PR González FH M CA Driemeier D Dall'alba MP Toxicity and genotoxicity evaluation of Passiflora alata Curtis (Passifloraceae) J Ethnopharmacol 2010 128 526 532 10.1016/j.jep.2009.09.037 19799991

[B28] Deng J Zhou Y Bai M Li H Li L Anxiolytic and sedative activities of Passiflora edulis f. flavicarpa J Ethnopharmacol 2010 128 148 153 10.1016/j.jep.2009.12.043 20051259

[B29] Masteikova R Bernatoniene J Bernatoniene R Velziene S Antiradical activities of the extract of Passiflora incarnata Acta Pol Pharm 2008 65 577 583 19051605

[B30] Dhawan K Kumar S Sharma A Anti-anxiety studies on extracts of Passiflora incarnata Linneaus J Ethnopharmacol 2001 78 165 170 10.1016/S0378-8741(01)00339-7 11694362

[B31] Dhawan K Kumar S Sharma A Comparative anxiolytic activity profile of various preparations of Passiflora incarnata linneaus: a comment on medicinal plants' standardization J Altern Complement Med 2002 8 283 291 10.1089/10755530260127970 12165186

[B32] Akhondzadeh S Naghavi HR Vazirian M Shayeganpour A Rashidi H Khani M Passionflower in the treatment of generalized anxiety: a pilot double-blind randomized controlled trial with oxazepam J Clin Pharm Ther 2001 26 363 367 10.1046/j.1365-2710.2001.00367.x 11679026

[B33] Movafegh A Alizadeh R Hajimohamadi F Esfehani F Nejatfar M Preoperative oral Passiflora incarnata reduces anxiety in ambulatory surgery patients: a double-blind, placebo-controlled study Anesth Analg 2008 106 1728 1732 10.1213/ane.0b013e318172c3f9 18499602

[B34] Bourin M Bougerol T Guitton B Broutin E A combination of plant extracts in the treatment of outpatients with adjustment disorder with anxious mood: controlled study versus placebo Fundamental 1997 11 127 132 10.1111/j.1472-8206.1997.tb00179.x 9107558

[B35] Cawte J Psychoactive substances of the South Seas: betel, kava and pituri Aust N Z J Psychiatry 1985 19 83 87 10.3109/00048678509158818 3890824

[B36] Singh YN Kava: an overview J Ethnopharmacol 1992 37 13 45 10.1016/0378-8741(92)90003-A 1453702

[B37] Bruner NR Anderson KG Discriminative-stimulus and time-course effects of kava-kava (Piper methysticum) in rats Pharmacol Biochem Behav 2009 92 297 303 10.1016/j.pbb.2008.12.017 19159643

[B38] Garrett KM Basmadjian G Khan IA Schaneberg BT Seale TW Extracts of kava (Piper methysticum) induce acute anxiolytic-like behavioral changes in mice Psychopharmacology (Berl) 2003 170 33 41 10.1007/s00213-003-1520-0 12845414

[B39] Singh YN Singh NN Therapeutic potential of kava in the treatment of anxiety disorders CNS Drugs 2002 16 731 743 10.2165/00023210-200216110-00002 12383029

[B40] Dinh LD Simmen U Bueter KB Bueter B Lundstrom K Schaffner W Interaction of various Piper methysticum cultivars with CNS receptors in vitro Planta Med 2001 67 306 311 10.1055/s-2001-14334 11458444

[B41] Yuan CS Dey L Wang A Mehendale S Xie JT Aung HH Ang-Lee MK Kavalactones and dihydrokavain modulate GABAergic activity in a rat gastric-brainstem preparation Planta Med 2002 68 1092 1096 10.1055/s-2002-36338 12494336

[B42] Volz HP Kieser M Kava-kava extract WS 1490 versus placebo in anxiety disorders--a randomized placebo-controlled 25-week outpatient trial Pharmacopsychiatry 1997 30 1 5 10.1055/s-2007-979474 9065962

[B43] Boerner RJ Sommer H Berger W Kuhn U Schmidt U Mannel M Kava-Kava extract LI 150 is as effective as Opipramol and Buspirone in Generalised Anxiety Disorder--an 8-week randomized, double-blind multi-centre clinical trial in 129 out-patients Phytomedicine 2003 10 38 49 10.1078/1433-187X-00309 12807341

[B44] Watkins LL K MC J RD Effect of kava extract on vagal cardiac control in generalized anxiety disorder: preliminary findings Journal of psychopharmacology (Oxford, England) 2001 15 283 286 10.1177/026988110101500407 11769822

[B45] Malsch U Kieser M Efficacy of kava-kava in the treatment of non-psychotic anxiety, following pretreatment with benzodiazepines Psychopharmacology (Berl) 2001 157 277 283 10.1007/s002130100792 11605083

[B46] Cagnacci A Arangino S Renzi A Zanni AL Malmusi S Volpe A Kava-Kava administration reduces anxiety in perimenopausal women Maturitas 2003 44 103 109 10.1016/S0378-5122(02)00317-1 12590005

[B47] Sarris J Kavanagh DJ Byrne G Bone KM Adams J Deed G The Kava Anxiety Depression Spectrum Study (KADSS): a randomized, placebo-controlled crossover trial using an aqueous extract of Piper methysticum Psychopharmacology (Berl) 2009 205 399 407 10.1007/s00213-009-1549-9 19430766

[B48] Scherer J Kava-kava extract in anxiety disorders: an outpatient observational study Advances in Therapy 1998 15 261 269 10186945

[B49] Boerner RJ Kava kava in the treatment of generalized anxiety disorder, simple phobia and specific social phobia Phytother Res 2001 15 646 647 10.1002/ptr.1006 11746854

[B50] Gastpar M Klimm HD Treatment of anxiety, tension and restlessness states with Kava special extract WS 1490 in general practice: a randomized placebo-controlled double-blind multicenter trial Phytomedicine 2003 10 631 639 10.1078/0944-7113-00369 14692723

[B51] Sarris J Kavanagh DJ Deed G Bone KM St. John's wort and Kava in treating major depressive disorder with comorbid anxiety: a randomised double-blind placebo-controlled pilot trial Hum Psychopharmacol 2009 24 41 48 10.1002/hup.994 19090505

[B52] Connor KM Davidson JR A placebo-controlled study of Kava kava in generalized anxiety disorder Int Clin Psychopharmacol 2002 17 185 188 10.1097/00004850-200207000-00005 12131602

[B53] Jacobs BP Bent S Tice JA Blackwell T Cummings SR An internet-based randomized, placebo-controlled trial of kava and valerian for anxiety and insomnia Medicine 2005 84 197 207 10.1097/01.md.0000172299.72364.95 16010204

[B54] Consumer Advisory: Kava-Containing Dietary Supplements May be Associated With Severe Liver Injury http://www.fda.gov/Food/ResourcesForYou/Consumers/ucm085482.htm

[B55] Teschke R Schwarzenboek A Akinci A Kava hepatotoxicity: a European view N Z Med J 2008 121 90 98 18841189

[B56] Linde K Ramirez G Mulrow CD Pauls A Weidenhammer W Melchart D St John's wort for depression - an overview and meta-analysis of randomised clinical trials BMJ 1996 313 8704532 10.1136/bmj.313.7052.253 PMC2351679

[B57] Butterweck V Mechanism of Action of St John's Wort in Depression: What is Known? CNS Drugs 2003 17 539 562 10.2165/00023210-200317080-00001 12775192

[B58] Woelk H Comparison of St John's wort and imipramine for treating depression: randomised controlled trial BMJ 2000 321 536 539 10.1136/bmj.321.7260.536 10968813 PMC27467

[B59] Kobak K Taylor L Bystrisky A Kohlenberg CJ Greist JH Tucker P Warner G Futterer R Vapnik T St John's wort versus placebo in obsessive-compulsive disorder: results from a double-blind study Int Clin Psychopharmacol 2005 20 299 304 10.1097/00004850-200511000-00003 16192837

[B60] Kobak K Taylor L Warnder G Futterer R St. John's wort versus placebo in social phobia: results from a placebo-controlled pilot study J Clin Psychopharmacol 2005 25 51 58 10.1097/01.jcp.0000150227.61501.00 15643100

[B61] Volz HP Murck H Kasper S Moller HJ St John's wort extract (LI 160) in somatoform disorders: results of a placebo-controlled trial Psychopharmacology 2002 164 294 300 10.1007/s00213-002-1171-6 12424553

[B62] Muller D Pfeil T von den Driesch V Treating depression comorbid with anxiety--results of an open, practice-oriented study with St. John's wort WS 5572 and valerian extract in high doses Phytomedicine 2003 10 25 30 10.1078/1433-187X-00305 12807339

[B63] Taylor L Kobak K An open-label trial of St. John's Wort (Hypericum perforatum) in obsessive-compulsive disorder J Clin Psychiatry 2000 61 575 578 10.4088/JCP.v61n0806 10982200

[B64] Huppert JD Schultz LT Foa EB Barlow DH Davidson JR Gorman JM Differential response to placebo among patients with social phobia, panic disorder, and obsessive-compulsive disorder Am J Psychiatry 2004 161 1485 1487 10.1176/appi.ajp.161.8.1485 15285978

[B65] Smriga M Torii K L-Lysine acts like a partial serotonin receptor 4 antagonist and inhibits serotonin-mediated intestinal pathologies and anxiety in rats Proc Natl Acad Sci USA 2003 100 15370 15375 10.1073/pnas.2436556100 14676321 PMC307574

[B66] Srinongkote S Smriga M Nakagawa K Toride Y A diet fortified with L-lysine and L-arginine reduces plasma cortisol and blocks anxiogenic response to transportation in pigs Nutr Neurosci 2003 6 283 289 10.1080/10284150310001614661 14609314

[B67] Jezova D Makatsori A Smriga M Morinaga Y Duncko R Subchronic treatment with amino acid mixture of L-lysine and L-arginine modifies neuroendocrine activation during psychosocial stress in subjects with high trait anxiety Nutr Neurosci 2005 8 155 160 10.1080/10284150500162937 16117182

[B68] Smriga M Ando T Akutsu M Furukawa Y Miwa K Morinaga Y Oral treatment with L-lysine and L-arginine reduces anxiety and basal cortisol levels in healthy humans Biomed Res 2007 28 85 90 10.2220/biomedres.28.85 17510493

[B69] Jezova D Makatsori A Duncko R Moncek F Jakubek M High trait anxiety in healthy subjects is associated with low neuroendocrine activity during psychosocial stress Prog Neuropsychopharmacol Biol Psychiatry 2004 28 1331 1336 10.1016/j.pnpbp.2004.08.005 15588760

[B70] Abraham GE Nutritional factors in the etiology of the premenstrual tension syndromes J Reprod Med 1983 28 446 464 6684167

[B71] Durlach J Pages N Bac P Bara M Guiet-Bara A Importance of magnesium depletion with hypofunction of the biological clock in the pathophysiology of headhaches with photophobia, sudden infant death and some clinical forms of multiple sclerosis Magnes Res 2004 17 314 326 15726907

[B72] Fromm L Heath DL Vink R Nimmo AJ Magnesium attenuates post-traumatic depression/anxiety following diffuse traumatic brain injury in rats J Am Coll Nutr 2004 23 529S 533S 15466958 10.1080/07315724.2004.10719396

[B73] German-Fattal M Lecerf F Sabbagh F Maurois P Durlach J Bac P Neuroprotective gene profile in the brain of magnesium-deficient mice Biomed Pharmacother 2008 62 264 272 10.1016/j.biopha.2008.02.007 18400454

[B74] Poleszak E Szewczyk B Kedzierska E Wlaz P Pilc A Nowak G Antidepressant- and anxiolytic-like activity of magnesium in mice Pharmacol Biochem Behav 2004 78 7 12 10.1016/j.pbb.2004.01.006 15159129

[B75] Carroll D Ring C Suter M Willemsen G The effects of an oral multivitamin combination with calcium, magnesium, and zinc on psychological well-being in healthy young male volunteers: a double-blind placebo-controlled trial Psychopharmacology (Berl) 2000 150 220 225 10.1007/s002130000406 10907676

[B76] De Souza MC Walker AF Robinson PA Bolland K A synergistic effect of a daily supplement for 1 month of 200 mg magnesium plus 50 mg vitamin B6 for the relief of anxiety-related premenstrual symptoms: a randomized, double-blind, crossover study J Womens Health Gend Based Med 2000 9 131 139 10.1089/152460900318623 10746516

[B77] Hanus M Lafon J Mathieu M Double-blind, randomised, placebo-controlled study to evaluate the efficacy and safety of a fixed combination containing two plant extracts (Crataegus oxyacantha and Eschscholtzia californica) and magnesium in mild-to-moderate anxiety disorders Curr Med Res Opin 2004 20 63 71 10.1185/030079903125002603 14741074

